# The role of functional health literacy in long-term treatment outcomes in psychosocial care for adolescents

**DOI:** 10.1007/s00787-019-01464-9

**Published:** 2020-01-10

**Authors:** L. Beukema, S. A. Reijneveld, M. Jager, J. Metselaar, A. F. de Winter

**Affiliations:** 1 Department of Health Sciences, University Medical Center Groningen, University of Groningen, Antonius Deusinglaan 1/FA10, 9713 AV Groningen, The Netherlands; 2 Department of Special Needs Education and Youth Care, University of Groningen, Groningen, The Netherlands

**Keywords:** Health literacy, Adolescent, Psychosocial problems, Psychosocial care, Treatment adherence, Longitudinal

## Abstract

Although among adolescents with psychosocial problems low health literacy may increase the risk of poor treatment outcomes, the contributing mechanisms within treatment remain unclear. A better understanding of these mechanisms could contribute to improved treatment processes and outcomes. This study aims to examine the relationship between functional health literacy, treatment processes (treatment adherence, learning processes), and treatment outcome (level of psychosocial problems) in adolescents in psychosocial care. We used data from a prospective cohort study among adolescents aged 12–18 (*N* = 390), collected in four successive measurements: at entry into care, and 3, 12, and 24 months thereafter. We used a mixed effect model to investigate the association between level of functional health literacy (adequate vs. inadequate) and treatment processes (treatment adherence, learning processes) and treatment outcome (level of psychosocial problems). Between adolescents with adequate and inadequate functional health literacy, we found no differences or change over time in adherence or learning processes. The level of psychosocial problems significantly declined over time (*β* = − 1.70, 95% CI [− 2.72, − 0.69], *p* = .001) to a similar degree in both groups, though, in all measurements, the level was consistently higher for adolescents with inadequate health literacy. We conclude that health literacy levels did not affect change in treatment processes nor in outcomes of psychosocial treatment. However, the consistently higher level of psychosocial problems among adolescents with inadequate health literacy suggests an unaddressed need in psychosocial care.

**Electronic supplementary material:**

The online version of this article (10.1007/s00787-019-01464-9) contains supplementary material, which is available to authorized users.

## Introduction

Psychosocial problems—emotional, behavioural, and social problems—are the third largest contributor to the global burden of disease in adolescents [1–3]. It is estimated that psychosocial problems affect up to 20% of children and adolescents [2, 4, 5] and that up to half of all adult psychopathologies have their roots in adolescence [4, 6]. Experiencing psychosocial problems in adolescence is related to a higher risk of poorer educational, social, occupational, and psychiatric outcomes later in life [2, 7, 8]. However, knowledge of factors that influence treatment outcomes in psychosocial care remains inadequate.

Client health literacy may be pivotal in the interpersonal communication between adolescent and professional, and in turn affect treatment outcomes [9]. The National Institute of Health (NIH) defines health literacy as the “degree to which individuals have the capacity to obtain, process and understand basic health information and services needed to make appropriate health decisions” [10]. At its core, health literacy is deemed dependent on individual capabilities, such as reading and writing skills, speaking and listening skills, and numeracy [11]. In line with this perspective, the current study focusses on functional health literacy, meaning that the basic literacy skills (i.e., reading and writing) needed in regular health care situations.

People with more adequate health literacy have skills and capabilities that enable them to act in a health enhancing way. Studies show that low health literacy is associated with more adverse health, less participation in treatment [12] as well as less adherence to treatment instructions, a lower desire to participate in decision-making, and less self-management [13]. In addition, low health literacy is associated with increased use of health care [14]. While most health literacy research has been focused on adults, researchers have started exploring the role of adolescent health literacy in health care. Nevertheless, evidence on the role of health literacy in psychosocial care for adolescents is lacking. Exploring this issue could help to improve future treatment processes and outcomes for adolescents in psychosocial care. This is especially important, because adolescents are in a developmental phase in which they are becoming increasingly autonomous and ready to make more of their own decisions regarding health and health care [14].

This study aimed to assess whether functional health literacy is associated with treatment processes (treatment adherence, learning processes) and treatment outcomes (level of psychosocial problems) in psychosocial care. We hypothesized that adolescents with lower functional health literacy participate less in treatment, experience fewer learning processes, and have less reduction in psychosocial problems 2 years after enrolment into psychosocial care.

## Methods

For our study, we used data from the Take Care study [15]. Take Care is a prospective cohort that included children and adolescents aged 4–18 years entering care for psychosocial problems. Take Care was designed to investigate trajectories and outcomes of care for youth with psychosocial problems in the Netherlands, and is part of C4Youth, the Collaborative Centre on Care for Children and Youth. The Medical Ethical Committee of the University Medical Centre of Groningen evaluated the design of the Take Care study, and approved it without requiring full assessment.

### Sample and procedure

Take Care included adolescents enrolled in psychosocial care organizations in the province of Groningen, the Northeast of the Netherlands, recruited between April 2011 and April 2013. They were recruited at three different types of care organizations: preventive child healthcare (PCH), child and adolescent social care (CASC), and child and adolescent mental healthcare (CAMH). Take Care consists of five measurements made during a 3-year follow-up, starting at entry into care and 3, 12, 24, and 36 months after the first questionnaire. A detailed description of the objectives, design, and measurements of Take Care can be found elsewhere [15]. This study appertains to data from the first four measurements.

Following their entry into care, adolescents and one parent/caregiver received information about the study and were invited to participate. Informed consent was obtained from the adolescent as well as from the parent, if the adolescent was younger than 16. Potential participants were excluded if they were older than 18, had severe mental retardation, were not living in one of the three northern Dutch provinces, or did not speak Dutch. The first questionnaire was sent shortly after entry into care and before the start of treatment.

The current study focused on adolescents (aged 12–18) who received care after the first measurement, for whom data on health literacy were available, and for whom data from the professional as well as one of the parents were also available (*N* = 390).

### Measures

We assessed health literacy, treatment processes (adherence, learning processes), treatment outcomes (level of psychosocial problems), and background variables and confounders (age, gender, educational level, ethnicity, and type of care in which adolescents were enrolled). Characteristics of the parent were also taken into account (educational level and level of health literacy).

Health literacy was measured at 3 months with three validated health literacy screening questions developed by Chew [16–19]. The questions have not been tested in an adolescent sample, but studies in other samples indicated high reliability [19, 20]. The three questions are: (1) “How often do you have someone help you read materials related to your care/treatment?” (2) “How confident are you filling out treatment-related forms by yourself?”, and (3) “How often do you have problems learning about your condition or symptoms because of difficulty understanding written information?” These questions were answered on a 5-point Likert scale. On item 1 and 2, the answer options were defined as: 1 ‘always’, 2 ‘often’, 3 ‘sometimes’, 4 ‘occasionally’, and 5 ‘never’. The answers options on item 3 were defined as: 1 ‘very much’, 2 ‘a lot’, 3 ‘somewhat’, 4 ‘a little’, and 5 ‘not at all’. After reversing the scores on the second question, a total score (range 3–15) was calculated by summing up the scores of the three questions, with a higher score indicating a higher level of health literacy. Due to the strongly skewed distribution, we chose to dichotomise the scores into a group with inadequate health literacy (score 11 or lower) and adequate health literacy (score 12 or higher). This cut-off point is based on previous studies, which used the same instrument [17, 20, 21], and in which comparable percentages of inadequate health literacy were found [22].

Treatment processes, i.e., treatment adherence and learning processes, were measured at 3 months, 1 year, and 2 years after intake. The assessment of these treatment processes was based on the previous studies using the Take Care data [9, 23]. Treatment adherence was measured by the agreement of the professional with the statement ‘the adolescent demonstrated adherence in between therapy sessions’, on a scale of 0–10 with higher values indicating stronger adherence. Learning processes were measured as improved understanding and improved confidence. Improved understanding was assessed by asking the professional as well as the adolescent how much they thought that the adolescent had learned so far due to psychosocial care. The specific questions were: “Please indicate how much you think the adolescent has learned from the treatment” and “Please indicate how much you have learned from the treatment you have received”. The questions included examples: better understanding of the problems and knowing how to handle difficult situations. Improved confidence was assessed by asking the professional as well as the adolescent whether the feelings of the adolescent had changed positively because of the psychosocial care. The specific questions were: “Please indicate how much you think the adolescents’ feelings have changed due to the treatment” and “Please indicate how much your feelings have changed due to the treatment you have received”. These questions also included examples: improved self-confidence, worrying less, and feeling less hopeless. Both learning processes were scored by the professionals as well as the adolescents themselves, and measured on a scale of 0–10 (0 ‘absolutely nothing’ and 10 ‘very much’). The distribution of the professional-rated and adolescent-rated scores was similar, although the professionals rated the improvement of the adolescents on average one point higher. To limit potential information bias, a total score was created by calculating the mean across the scores of the professional and adolescent per individual case for both learning processes. Since major discrepancies between adolescents’ and professionals’ scores were rare, convergence of their scores was deemed appropriate and leading to more accurate estimates [24].

Treatment outcomes, i.e., the level of psychosocial problems, were measured at entry into care, 3 months, 1 year, and 2 years after intake. Psychosocial problems were assessed with the Dutch self-report and parent-report versions of the Strengths and Difficulties Questionnaire (SDQ) [25–28]. The SDQ consists of 25 items, measuring internalizing [Cronbach’s *α* parents = 0.78 (T1, T2, T3), adolescents = 0.75 (T1), 0.76 (T2), and 0.73 (T3)] and externalizing problems [Cronbach’s *α* parents = 0.83 (T1, T2, T3), adolescents = 0.74 (T1), 0.76 (T2), and 0.73 (T3)] [29]. The total difficulties score (TDS) ranges from 0 to 40, with higher scores indicating more problems. Studies have shown that detection of adolescent psychopathology improves by combining multi-informant data [30–32], and the TDSs of adolescents and their parents were added and divided by two, resulting in a mean TDS across two informants, and following the same procedure as previously applied to this data [9].

### Background characteristics and confounders

Background characteristics and confounders that were included regarded the adolescents’ gender, age, educational level, ethnicity, type of care, parental health literacy, and parental educational level. Adolescent educational level represented the level of current education and not the highest obtained diploma, because most adolescents were still in school. Educational level was categorised as (1) low (practical training, pre-vocational secondary education, special needs education, or lower levels of secondary vocational education), (2) medium/high (senior general secondary education, pre-university secondary education, higher levels of secondary vocational education, or higher professional education), or (3) undetermined (still at primary school or unknown). Ethnicity was defined as non-Dutch if the adolescent or at least one biological parent had been born outside the Netherlands. Type of care that an adolescent was enrolled in was either preventive child healthcare (PCH), child and adolescent social care (CASC), or child and adolescent mental healthcare (CAMH). Parental health literacy was measured and calculated in the same way as that of the adolescent, as described above. Parental education level was defined by the highest diploma obtained and categorised in the same way as the educational level of the adolescent.

### Statistical analyses

First, we described the background characteristics and covariates of the sample, and the level of health literacy (adequate vs. inadequate). Second, we analysed the association between level of health literacy (adequate vs. inadequate), treatment processes (adherence, improved understanding, improved confidence), and treatment outcomes (level of psychosocial problems), using a mixed effect model. This is a fitting approach to investigate individual change over time, inserting the longitudinal data in a multilevel model with measurements per individual nested within individuals [33, 34]. The data were analysed using a mixed effect model with random intercepts and fixed slopes. To choose best model, the Bayesian information criterion (BIC) was used. BIC is based on the log-likelihood of the model and the number of parameters in the model. As is standard, the model with the lowest BIC was used. The best-fitting model was based on restricted maximum-likelihood (REML) estimation and an unstructured covariance structure. REML gives the most accurate estimates of random variances and an unstructured covariance structure is preferable when estimating variances of random effects. Time was included in the model as a continuous variable (baseline = 0; 3 months = 0.25; 1 year = 1; 2 years = 2). For every outcome variable, we modelled two levels: the level 1 model includes the repeated measurements over time and does not include predictors. This model focuses on the main trend of the outcome variable over time. In the level 2 model, the predictors are added to test whether they are associated with the change over time found in the level 1 model. For example, for the outcome variable treatment adherence, we first modelled the main trend of treatment adherence over time (level 1 model), and then, we added the predictors (health literacy and the confounders) to test the level 2 model. We repeated these steps for the other outcome variable (improved understanding, improved confidence, and psychosocial problems). All statistical analyses were performed using SPSS for Windows (SPSS Inc., Chicago, IL, USA, version 25.0).

## Results

### Background characteristics

Adolescent, family, and care-related characteristics are presented in Table 1. The majority of the sample had adequate health literacy, were female and had Dutch ethnicity. In addition, most adolescents received care from a CAMH organization, followed by CASC and PCH. Level of health literacy was significantly associated with the level of psychosocial problems at baseline (*d* = 0.33, *p* = 0.002), as well as with educational level of the adolescent (*V* = 0.154, *p* = 0.016), and with age (*d* = 0.34, *p* = 0.001).

**Table 1:** Characteristics of the sample at baseline, by level of health literacy

	Total (*N* = 390)	Health literacy		*P*^a^
		Inadequate	Adequate	
		(*N* = 158, 40.5%)	(*N* = 232, 59.5%)	
	*N* (%)^b^	*N* (%)^b^	*N* (%)^b^	
Adolescent characteristics				
Female	223 (57.2)	92 (58.2)	131 (56.5)	0.73
Educational level				**0.016**
Low	206 (52.8)	88 (62.9)	118 (56.5)	
Medium/high	104 (26.7)	31 (22.1)	73 (34.9)	
Undetermined	39 (10.0)	21 (15.0)	18 (8.6)	
Dutch ethnicity	311 (79.1)	129 (90.2)	182 (87.5)	0.43
	M (SD)^c^	M (SD)^c^	M (SD)^c^	
Age	15.0 (1.8)	14.7 (1.7)	15.3 (1.8)	**0.001**
Psychosocial problems at baseline	15.0 (5.0)	16.0 (5.2)	14.3 (4.8)	**0.002**
Family characteristics				
Parent health literacy				0.05
Inadequate	121 (31.0)	85 (59.4)	143 (69.4)	
Adequate	228 (58.5)	58 (40.6)	63 (30.6)	
Educational level mother				0.78
Low	149 (38.2)	64 (47.8)	85 (43.8)	
Medium/high	174 (44.6)	68 (50.7)	106 (54.6)	
Undetermined	5 (1.3)	2 (1.5)	3 (1.5)	
Educational level father				0.34
Low	144 (36.9)	62 (45.9)	82 (40.9)	
Medium/high	155 (39.7)	62 (45.9)	93 (46.3)	
Undetermined	37 (9.5)	11 (8.1)	26 (12.9)	
Care characteristics				
Type of care				0.63
Preventive	38 (9.7)	18 (11.4)	20 (8.6)	
Social	80 (20.5)	33 (20.9)	47 (20.3)	
Mental	272 (69.7)	107 (67.7)	165 (71.1)	

*M* mean, *SD* standard deviation

^a^Chi-square tests were used for categorical variables (gender, adolescents’ educational level, ethnicity, parents’ educational level, parent health literacy, and care type) and *t* tests for continuous variables (age and psychosocial problems)

^b^ Numbers do not always add up to *N* = 390 due to missing values

### Health literacy level, treatment processes, and outcomes

Table 2 shows the results of the mixed model analysis, in which the columns represent the results per outcome. The analysis on the outcome variable ‘treatment adherence’ showed no change in treatment adherence over time (*β* = 0.15, 95% CI [− 0.08, 0.38], *p* = 0.21). The analyses on the outcome variables ‘improved understanding’ (*β* = 0.27, 95% CI [0.10, 0.45], *p* = 0.003) and ‘improved confidence’ (*β* = 0.26, 95% CI [0.07, 0.45], *p* = 0.007) showed a significant, yet slight, increase over time. Analysis further showed that neither outcome was significantly associated with health literacy, not as an effect and not as interaction. This means that the scores on ‘improved understanding’ and ‘improved confidence’ did not differ between adolescents with inadequate and adequate health literacy over time. In addition, the slight increases in ‘improved understanding’ and ‘improved confidence’ were also not associated with the level of health literacy.

**Table 2:** Associations of health literacy with treatment processes (adherence, improved understanding, and improved confidence) and treatment outcomes (psychosocial problems): results of mixed model analysis with parameter estimates (*β*), 95% confidence intervals (CI), and *p* values

	Adherence		Improved understanding		Improved confidence		Psychosocial problems	
	*β* (95% CI)	*p*	*β* (95% CI)	*p*	*β* (95% CI)	*p*	*β* (95% CI)	*p*
Time	0.15 (−0.08, 0.38)	0.21	0.27 (0.10, 0.45)	**0.003**	0.26 (0.07, 0.45)	**0.007**	−1.70 (−1.94, −1.46)	**< 0.001**
Health literacy	0.43 (−0.27, 1.14)	0.23	0.37 (−0.15, 0.90)	0.16	0.41 (−0.16, 0.99)	0.16	−1.70 (−2.72, −0.69)	**.001**
Time*Health literacy	–^a^	–^a^	−0.16 (−0.57, 0.24)	0.42	−0.36 (−0.81, 0.10)	0.12	0.22 (−0.30, 0.73)	0.40

Time was included as a continuous variable with the values: baseline = 0; 3 months = 0.25; 1 year = 1; 2 years = 2. All analyses were adjusted for age, gender, education level, ethnicity of the adolescent, type of care, and parental health literacy

^a^No parameter estimate is given as the main effect of time is not significant

The level of psychosocial problems declined significantly over time (*β* = − 1.70, 95% CI [− 1.94, − 1.46], *p* < 0.001). Further analysis showed a significant difference in level of psychosocial problems between adolescents with adequate and inadequate health literacy (*β* = − 1.70, 95% CI [− 2.72, − 0.69], *p* = 0.001). However, health literacy level was not associated with the degree of change in psychosocial problems over time; i.e., both adolescents with adequate and inadequate health literacy showed the same decline in problems (*β* = 0.22, 95% CI [− 0.30, 0.73], *p* = 0.40). Thus, the difference in level of psychosocial problems, observed at the first measurement, persisted over time.

Since educational level was associated with level of health literacy, we repeated the analyses without adjusting for educational level. This lead to similar results.

## Discussion

This is the first longitudinal study to examine health literacy of adolescents in psychosocial care. We found no association between health literacy level and treatment adherence or learning processes. During treatment, levels of psychosocial problems for adolescents with both inadequate and adequate health literacy decreased to a similar degree, but remained higher for adolescents with inadequate health literacy.

We found no association between health literacy level and changes in treatment adherence or learning processes. These results refute our hypothesis that adolescents with inadequate health literacy are likely to participate less in treatment and experience fewer learning processes. In regard to treatment adherence, previous studies have shown mixed or even weak evidence on the relationship between health literacy and adherence [35–37]. To our knowledge, no previous research has been done on the association between health literacy and improved understanding and confidence. However, previous studies did show positive associations between adequate health literacy and higher levels of health knowledge, positive beliefs related to health, and self-efficacy [38, 39]. Our results could indicate that in dealing with adolescents with different levels of health literacy, professionals have adequately tailored their treatment. Another explanation may be that most health literacy studies focussed on the medical care setting. Health literacy level may simply not play a role in adherence and learning processes in psychosocial care settings. Third, our measure of health literacy may have been insufficiently sensitive for use in the psychosocial care setting. Finally, missing data on the third and fourth measurement for the outcome variables adherence and learning processes may have resulted in decreased power to detect differences.

We further found that, over the 2-year period following entry into care, levels of psychosocial problems decreased to a similar degree for adolescents with both inadequate and adequate health literacy. It is noteworthy that, at entry into care, adolescents with inadequate health literacy had a significantly higher level of psychosocial problems compared to adolescents with adequate health literacy, and that this difference in psychosocial problems remained the same over the following 2 years. This suggests that adolescents with inadequate health literacy already enter care at a disadvantage and are not able to catch up during a treatment period of 2 years. Other factors which we did not take into account, such as situational determinants (e.g., social support, family, and peer influences), may also play a role here [38]. It should be noted, however, that the difference in level of psychosocial problems, between adolescents with adequate and inadequate health literacy, did not change during treatment. The professionals in this study may have been sufficiently patient-centred to cope with the low health literacy in this group, thus preventing the worsening of relative outcomes over time, as suggested by the other studies [40]. Adequate patient-centred communication, an important determinant of treatment outcomes in psychosocial care for adolescents, may have contributed here [9]. Patient-centred communication strategies may be especially helpful for patients with an inadequate literacy level [41].

### Strengths and limitations

This study has major strengths, one being that we were able to assess changes over time and the relationship between health literacy levels and long-term outcomes of psychosocial care, using a representative sample of adolescents entering psychosocial care in one catchment area during a 2-year period. Another strength is that we used two groups of informants: adolescents and their parents for the measurements of psychosocial problems, and adolescents and professionals for measurement of learning processes. Combining different informants, and thereby different perspectives, potentially results in a more objective and stable measure of psychopathology [30, 42].

Several limitations of the study must also be considered. First, regarding our measure of health literacy. The health literacy questions used in this study have not yet been validated for use with adolescents, which casts some uncertainty on the discrimination between those with adequate and inadequate health literacy. However, studies on adults show its validity in general to be high, reducing the likelihood of considerable measurement error. Second, the current study documents the relationship between functional health literacy and treatment outcomes, while insight into other aspects of health literacy may be of importance as well, such as communicative/interactive health literacy, defined as the cognitive and social skills necessary to actively communicate in regard to health information, and critical health literacy, defined as the ability to act on health information [11, 43]. Third, selection bias may have been introduced through the sampling method. However, differences in background characteristics between respondents and non-respondents at baseline were small [15].

### Implications

This study contributes to the growing research literature on health literacy in adolescents. We found that during treatment, levels of psychosocial problems for adolescents with both inadequate and adequate health literacy decreased to a similar degree, and remained higher for adolescents with inadequate health literacy. This finding suggests an unmet need: adolescents with inadequate health literacy already enter care at a disadvantage. They improve, but do not catch up during treatment compared to those with adequate health literacy.

Our findings further show a need for better ways to measure adolescent health literacy. This applies both to the validity of current measures, as well as their augmentation to include a greater range of aspects of health literacy. This may imply a need for a health literacy measure that is more suitable for the psychosocial care setting. For example, mental health literacy encompasses mental health knowledge, attitudes of stigma, and help-seeking [44]. However, the functional health literacy skills, as investigated in the current study, may also play a role in the psychosocial care setting. This implies a need for further research on the concept and instruments of health literacy and mental health literacy in the psychosocial care setting [45, 46].

Our results also highlight the need for further study on how to reduce health differences between adolescents with inadequate and adequate health literacy in psychosocial care, such as exploring interventions to decrease the rates of psychosocial problems in adolescents with inadequate health literacy. Further exploration into interventions to improve health literacy among these adolescents may also improve their treatment outcomes. Previous studies in other, more susceptible, patient groups have indicated that health literacy interventions are effective [12].

## Electronic supplementary material

Below is the link to the electronic supplementary material.

Supplementary file1 (DOCX 17 kb)

## Data Availability

The data that support the findings of this study are available on request.
