# Supplementary material for: The role of functional health literacy in long-term treatment outcomes in psychosocial care for adolescents
Source: Eur Child Adolesc Psychiatry. 2020 Jan 10;29(11):1547–54. doi: 10.1007/s00787-019-01464-9 (PMC7595965; doi:10.1007/s00787-019-01464-9)
Supplement: Supplementary file 1 — Supplementary file1 (DOCX 17 kb) [file 787_2019_1464_MOESM1_ESM.docx]

**Supplementary material**: Syntax of the mixed model analysis*European Child and Adolescent Psychiatry*

The role of functional health literacy in long-term treatment outcomes in psychosocial care for adolescents

Beukema, L.^1*^, Reijneveld, S.A.^1^, Jager, M.^1^, Metselaar, J.^2^, De Winter, A.F.^1^

^1^ Department of Health Sciences, University Medical Center Groningen, University of Groningen, Antonius Deusinglaan 1/FA10, 9713 AV Groningen, The Netherlands

^2^ Department of Special Needs Education and Youth Care, University of Groningen, Groningen, The Netherlands

* Correspondence: l.beukema@umcg.nl

Timeyears = Time continuous (0 = T1/baseline, 0.25 = T2/3 months, 1 = T3/1 year, 2 = T4/2 years)

HL = Health literacy

GEN3cMi1 = Gender

AGE = Age

ETN = Ethnicity

EDULEVEL = Educational level

CareTypeT1 = Type of care

HLgroupparent = level of health literacy parent

ADH= Adherence

UND = Improved understanding

CON = Improved confidence

SDQ = Psychosocial problems

**MM with outcome 1: ADHERENCE (ADH)*

*1A. level-1 model incl time continuous*

MIXED ADH WITH Timeyears

/CRITERIA=CIN(95) MXITER(1000) MXSTEP(100) SCORING(1) SINGULAR(0.000000000001) HCONVERGE(0, ABSOLUTE) LCONVERGE(0, ABSOLUTE) PCONVERGE(0.000001, ABSOLUTE)

/FIXED=Timeyears | SSTYPE(3)

/METHOD=REML

/PRINT=SOLUTION TESTCOV

/RANDOM=INTERCEPT Timeyears | SUBJECT(IDkindMerge) COVTYPE(UN)

/SAVE=RESID.

**1B. level-2 model adding predictors*

MIXED ADH WITH Timeyears HLgroup GEN3cMi1 AGE ETN EDULEVEL CareTypeT1 HLgroupparent

/CRITERIA=CIN(95) MXITER(1000) MXSTEP(100) SCORING(1) SINGULAR(0.000000000001) HCONVERGE(0, ABSOLUTE) LCONVERGE(0, ABSOLUTE) PCONVERGE(0.000001, ABSOLUTE)

/FIXED=Timeyears HLgroup GEN3cMi1 AGE ETN EDULEVEL CareTypeT1 HLgroupparent HLgroup*Timeyears GEN3cMi1*Timeyears AGE*Timeyears ETN*Timeyears EDULEVEL*Timeyears CareTypeT1*Timeyears HLgroupparent*Timeyears | SSTYPE(3)

/METHOD=REML

/PRINT=SOLUTION TESTCOV

/RANDOM=INTERCEPT Timeyears | SUBJECT(IDkindMerge) COVTYPE(UN).

**MM with outcome 2: Improved Understanding (UND)*

*2A. level-1 model incl time continuous*

MIXED UND WITH Timeyears

/CRITERIA=CIN(95) MXITER(1000) MXSTEP(100) SCORING(1) SINGULAR(0.000000000001) HCONVERGE(0, ABSOLUTE) LCONVERGE(0, ABSOLUTE) PCONVERGE(0.000001, ABSOLUTE)

/FIXED=Timeyears | SSTYPE(3)

/METHOD=REML

/PRINT=SOLUTION TESTCOV

/RANDOM=INTERCEPT Timeyears | SUBJECT(IDkindMerge) COVTYPE(UN)

/SAVE=RESID.

** 2B. level-2 model adding predictors*

MIXED UND WITH Timeyears HLgroup GEN3cMi1 AGE ETN EDULEVEL CareTypeT1 HLgroupparent

/CRITERIA=CIN(95) MXITER(1000) MXSTEP(100) SCORING(1) SINGULAR(0.000000000001) HCONVERGE(0, ABSOLUTE) LCONVERGE(0, ABSOLUTE) PCONVERGE(0.000001, ABSOLUTE)

/FIXED=Timeyears HLgroup GEN3cMi1 AGE ETN EDULEVEL CareTypeT1 HLgroupparent HLgroup*Timeyears GEN3cMi1*Timeyears AGE*Timeyears ETN*Timeyears EDULEVEL*Timeyears CareTypeT1*Timeyears HLgroupparent*Timeyears | SSTYPE(3)

/METHOD=REML

/PRINT=SOLUTION TESTCOV

/RANDOM=INTERCEPT Timeyears | SUBJECT(IDkindMerge) COVTYPE(UN).

**MM with outcome 3: Improved Confidence (CON)*

*3A. level-1 model incl time continuous*

MIXED CON WITH Timeyears

/CRITERIA=CIN(95) MXITER(1000) MXSTEP(100) SCORING(1) SINGULAR(0.000000000001) HCONVERGE(0, ABSOLUTE) LCONVERGE(0, ABSOLUTE) PCONVERGE(0.000001, ABSOLUTE)

/FIXED=Timeyears | SSTYPE(3)

/METHOD=REML

/PRINT=SOLUTION TESTCOV

/RANDOM=INTERCEPT Timeyears | SUBJECT(IDkindMerge) COVTYPE(UN)

/SAVE=RESID.

** 3B. level-2 model adding predictors*

MIXED CON WITH Timeyears HLgroup GEN3cMi1 AGE ETN EDULEVEL CareTypeT1 HLgroupparent

/CRITERIA=CIN(95) MXITER(1000) MXSTEP(100) SCORING(1) SINGULAR(0.000000000001) HCONVERGE(0, ABSOLUTE) LCONVERGE(0, ABSOLUTE) PCONVERGE(0.000001, ABSOLUTE)

/FIXED=Timeyears HLgroup GEN3cMi1 AGE ETN EDULEVEL CareTypeT1 HLgroupparent HLgroup*Timeyears GEN3cMi1*Timeyears AGE*Timeyears ETN*Timeyears EDULEVEL*Timeyears CareTypeT1*Timeyears HLgroupparent*Timeyears | SSTYPE(3)

/METHOD=REML

/PRINT=SOLUTION TESTCOV

/RANDOM=INTERCEPT Timeyears | SUBJECT(IDkindMerge) COVTYPE(UN).

**MM with outcome 4: Psychosociale problems (SDQ)*

*4A. level-1 model incl time continuous*

MIXED SDQ WITH Timeyears

/CRITERIA=CIN(95) MXITER(1000) MXSTEP(100) SCORING(1) SINGULAR(0.000000000001) HCONVERGE(0, ABSOLUTE) LCONVERGE(0, ABSOLUTE) PCONVERGE(0.000001, ABSOLUTE)

/FIXED=Timeyears | SSTYPE(3)

/METHOD=REML

/PRINT=SOLUTION TESTCOV

/RANDOM=INTERCEPT Timeyears | SUBJECT(IDkindMerge) COVTYPE(UN)

/SAVE=RESID.

**4B. level-2 model adding predictors*

MIXED SDQ WITH Timeyears HLgroup GEN3cMi1 AGE ETN EDULEVEL CareTypeT1 HLgroupparent

/CRITERIA=CIN(95) MXITER(1000) MXSTEP(100) SCORING(1) SINGULAR(0.000000000001) HCONVERGE(0, ABSOLUTE) LCONVERGE(0, ABSOLUTE) PCONVERGE(0.000001, ABSOLUTE)

/FIXED=Timeyears HLgroup GEN3cMi1 AGE ETN EDULEVEL CareTypeT1 HLgroupparent HLgroup*Timeyears GEN3cMi1*Timeyears AGE*Timeyears ETN*Timeyears EDULEVEL*Timeyears CareTypeT1*Timeyears HLgroupparent*Timeyears | SSTYPE(3)

/METHOD=REML

/PRINT=SOLUTION TESTCOV

/RANDOM=INTERCEPT Timeyears | SUBJECT(IDkindMerge) COVTYPE(UN).

**Same models without controlling for EDULEVEL.*

**MM with outcome 1: ADHERENCE (ADH)*

*1A. level-1 model incl time continuous*

MIXED ADH WITH Timeyears

/CRITERIA=CIN(95) MXITER(1000) MXSTEP(100) SCORING(1) SINGULAR(0.000000000001) HCONVERGE(0, ABSOLUTE) LCONVERGE(0, ABSOLUTE) PCONVERGE(0.000001, ABSOLUTE)

/FIXED=Timeyears | SSTYPE(3)

/METHOD=REML

/PRINT=SOLUTION TESTCOV

/RANDOM=INTERCEPT Timeyears | SUBJECT(IDkindMerge) COVTYPE(UN)

/SAVE=RESID.

**1B. level-2 model adding predictors*

MIXED ADH WITH Timeyears HLgroup GEN3cMi1 AGE ETN CareTypeT1 HLgroupparent

/CRITERIA=CIN(95) MXITER(1000) MXSTEP(100) SCORING(1) SINGULAR(0.000000000001) HCONVERGE(0, ABSOLUTE) LCONVERGE(0, ABSOLUTE) PCONVERGE(0.000001, ABSOLUTE)

/FIXED=Timeyears HLgroup GEN3cMi1 AGE ETN CareTypeT1 HLgroupparent HLgroup*Timeyears GEN3cMi1*Timeyears AGE*Timeyears ETN*Timeyears CareTypeT1*Timeyears HLgroupparent*Timeyears | SSTYPE(3)

/METHOD=REML

/PRINT=SOLUTION TESTCOV

/RANDOM=INTERCEPT Timeyears | SUBJECT(IDkindMerge) COVTYPE(UN).

**MM with outcome 2: Improved Understanding (UND)*

*2A. level-1 model incl time continuous*

MIXED UND WITH Timeyears

/CRITERIA=CIN(95) MXITER(1000) MXSTEP(100) SCORING(1) SINGULAR(0.000000000001) HCONVERGE(0, ABSOLUTE) LCONVERGE(0, ABSOLUTE) PCONVERGE(0.000001, ABSOLUTE)

/FIXED=Timeyears | SSTYPE(3)

/METHOD=REML

/PRINT=SOLUTION TESTCOV

/RANDOM=INTERCEPT Timeyears | SUBJECT(IDkindMerge) COVTYPE(UN)

/SAVE=RESID.

** 2B. level-2 model adding predictors*

MIXED UND WITH Timeyears HLgroup GEN3cMi1 AGE ETN CareTypeT1 HLgroupparent

/CRITERIA=CIN(95) MXITER(1000) MXSTEP(100) SCORING(1) SINGULAR(0.000000000001) HCONVERGE(0, ABSOLUTE) LCONVERGE(0, ABSOLUTE) PCONVERGE(0.000001, ABSOLUTE)

/FIXED=Timeyears HLgroup GEN3cMi1 AGE ETN CareTypeT1 HLgroupparent HLgroup*Timeyears GEN3cMi1*Timeyears AGE*Timeyears ETN*Timeyears CareTypeT1*Timeyears HLgroupparent*Timeyears | SSTYPE(3)

/METHOD=REML

/PRINT=SOLUTION TESTCOV

/RANDOM=INTERCEPT Timeyears | SUBJECT(IDkindMerge) COVTYPE(UN).

**MM with outcome 3: Improved Confidence (CON)*

*3A. level-1 model incl time continuous*

MIXED CON WITH Timeyears

/CRITERIA=CIN(95) MXITER(1000) MXSTEP(100) SCORING(1) SINGULAR(0.000000000001) HCONVERGE(0, ABSOLUTE) LCONVERGE(0, ABSOLUTE) PCONVERGE(0.000001, ABSOLUTE)

/FIXED=Timeyears | SSTYPE(3)

/METHOD=REML

/PRINT=SOLUTION TESTCOV

/RANDOM=INTERCEPT Timeyears | SUBJECT(IDkindMerge) COVTYPE(UN)

/SAVE=RESID.

** 3B. level-2 model adding predictors*

MIXED CON WITH Timeyears HLgroup GEN3cMi1 AGE ETN CareTypeT1 HLgroupparent

/CRITERIA=CIN(95) MXITER(1000) MXSTEP(100) SCORING(1) SINGULAR(0.000000000001) HCONVERGE(0, ABSOLUTE) LCONVERGE(0, ABSOLUTE) PCONVERGE(0.000001, ABSOLUTE)

/FIXED=Timeyears HLgroup GEN3cMi1 AGE ETN CareTypeT1 HLgroupparent HLgroup*Timeyears GEN3cMi1*Timeyears AGE*Timeyears ETN*Timeyears CareTypeT1*Timeyears HLgroupparent*Timeyears | SSTYPE(3)

/METHOD=REML

/PRINT=SOLUTION TESTCOV

/RANDOM=INTERCEPT Timeyears | SUBJECT(IDkindMerge) COVTYPE(UN).

**MM with outcome 4: Psychosociale problems (SDQ)*

*4A. level-1 model incl time continuous*

MIXED SDQ WITH Timeyears

/CRITERIA=CIN(95) MXITER(1000) MXSTEP(100) SCORING(1) SINGULAR(0.000000000001) HCONVERGE(0, ABSOLUTE) LCONVERGE(0, ABSOLUTE) PCONVERGE(0.000001, ABSOLUTE)

/FIXED=Timeyears | SSTYPE(3)

/METHOD=REML

/PRINT=SOLUTION TESTCOV

/RANDOM=INTERCEPT Timeyears | SUBJECT(IDkindMerge) COVTYPE(UN)

/SAVE=RESID.

**4B. level-2 model adding predictors*

MIXED SDQ WITH Timeyears HLgroup GEN3cMi1 AGE ETN CareTypeT1 HLgroupparent

/CRITERIA=CIN(95) MXITER(1000) MXSTEP(100) SCORING(1) SINGULAR(0.000000000001) HCONVERGE(0, ABSOLUTE) LCONVERGE(0, ABSOLUTE) PCONVERGE(0.000001, ABSOLUTE)

/FIXED=Timeyears HLgroup GEN3cMi1 AGE ETN CareTypeT1 HLgroupparent HLgroup*Timeyears GEN3cMi1*Timeyears AGE*Timeyears ETN*Timeyears CareTypeT1*Timeyears HLgroupparent*Timeyears | SSTYPE(3)

/METHOD=REML

/PRINT=SOLUTION TESTCOV

/RANDOM=INTERCEPT Timeyears | SUBJECT(IDkindMerge) COVTYPE(UN).
